# Derivation of oligonucleotide barcodes that are absent from natural sequences

**DOI:** 10.1093/bioadv/vbag251

**Published:** 2026-08-28

**Authors:** Michail Patsakis, Kimonas Provatas, Ioannis Mouratidis, Ilias Georgakopoulos-Soares

**Affiliations:** Division of Pharmacology and Toxicology, College of Pharmacy, The University of Texas at Austin, Austin, TX 78712, United States; Dell Pediatric Research Institute, Austin, TX 78723, United States; Department of Computer Science, College of Natural Sciences, The University of Texas at Austin, Austin, TX 78712, United States; Division of Pharmacology and Toxicology, College of Pharmacy, The University of Texas at Austin, Austin, TX 78712, United States; Dell Pediatric Research Institute, Austin, TX 78723, United States; Department of Computer Science, College of Natural Sciences, The University of Texas at Austin, Austin, TX 78712, United States; Division of Pharmacology and Toxicology, College of Pharmacy, The University of Texas at Austin, Austin, TX 78712, United States; Dell Pediatric Research Institute, Austin, TX 78723, United States; Division of Pharmacology and Toxicology, College of Pharmacy, The University of Texas at Austin, Austin, TX 78712, United States; Dell Pediatric Research Institute, Austin, TX 78723, United States; Department of Life Sciences, School of Sciences, European University Cyprus, Nicosia 1516, Cyprus

## Abstract

DNA barcodes are short synthetic sequences used to uniquely identify target molecules, samples, or objects, and are essential for a wide range of biotechnology applications including high-throughput sequencing, lineage tracing, genetic screens, massively parallel reporter assays, and DNA-based data storage. However, randomly generated barcodes are prone to off-target hybridization and cross-reactivity with endogenous biological sequences, which can compromise experimental specificity and interpretation. Barcodes derived from sequences that are entirely absent from natural genomes, termed DNA primes, offer an ideal solution by eliminating the possibility of unintended interactions with biological material. Here, we present barcodesDB, a comprehensive database of synthetic DNA barcodes systematically identified by scanning 403 199 complete organismal genome assemblies across the tree of life, together with 215 Gbp of raw metagenomic sequencing reads spanning marine, soil, polar and host-associated environments. These barcodes are absent, on either strand, from every assembly and sequencing read examined in the release described, providing maximal specificity and minimizing cross-reactivity for downstream applications. We provide an open-access web application that enables researchers to search for barcodes satisfying user-defined constraints, including GC content and substring requirements, and to query whether candidate sequences occur in nature. This resource supports robust and scalable barcode design for diverse experimental and applied contexts. Our database is publicly available at: https://barcodesdb.com/.

## 1. Introduction

Advances in DNA synthesis technologies, together with declining costs, enable the cost-effective production of large pools of synthetic oligonucleotides. A DNA barcode is a sequence utilized to identify a specific target molecule or object. DNA barcoding is essential for a number of biotechnology applications and can significantly enhance the efficiency of high throughput experiments, enabling studies that would otherwise be excessively time-consuming, expensive, and challenging to perform.

Incorporation of barcodes is employed in various high-throughput experimental setups, including lineage tracing (McKenna *et al.* 2016, Zhang *et al.* 2024), genetic screens (Eason *et al.* 2004, Smith *et al.* 2009), next generation sequencing to multiplex samples (Costea *et al.* 2013), and in massively parallel reporter assays (Lee *et al.* 2021). Libraries of molecules, such as shRNAs and CRISPR guides, are tagged with unique barcodes to facilitate these studies (Lyons *et al.* 2017). Barcodes can also be utilized to identify and quantify species within complex microbial or ecological communities in metagenomics and environmental DNA studies (Qian *et al.* 2020). Furthermore, conventional digital storage has information density limitations and short life expectancy (Anžel *et al.* 2021). DNA storage offers an alternative, leveraging the exceptionally high information density and long-term stability of DNA molecules (Goswami *et al.* 2013, Erlich and Zielinski 2017). These same properties enable the use of synthetic DNA barcodes as long-term, information-rich labels, allowing large numbers of samples to be uniquely tagged and tracked using minimal material (Goswami *et al.* 2013).

Barcodes can be created either randomly or as part of rationally designed DNA barcode libraries. However, random barcodes in large libraries are prone to issues such as off-target hybridization and cross-reactivity with biological sequences (Gowri *et al.* 2024). The rational design of synthetic barcodes integrates multiple constraints to optimize performance and mitigate these limitations. These criteria include minimizing sequencing errors (Buschmann and Bystrykh 2013), avoiding cross-reactivity with natural sequences and with other barcodes (Erlich and Zielinski 2017), and excluding specific sequences such as restriction sites (Storm and Jensen 2018), repeating subsequences, and homopolymer stretches (Fazekas *et al.* 2010). Additional considerations involve preventing the formation of repeats and secondary structures, while ensuring uniform melting temperatures and adhering to GC content constraints (Johnson *et al.* 2023).

*k*-mers are short sequences of length *k*, and are fundamental units in various biological analyses and applications (Moeckel *et al.* 2024). Nullomers are the subset of *k*-mers that are absent from a genome, and primes are the set of *k*-mers absent from every genome (Hampikian and Andersen 2007, Georgakopoulos-Soares *et al.* 2021, Koulouras and Frith 2021). Previous work has shown the utility of nullomers and primes in developing DNA barcodes with heightened specificity (Goswami *et al.* 2013). Here, we systematically scan all complete organismal genomes available in NCBI, amassing 4 03 199 complete genome assemblies of organisms across the tree of life, which we supplement with 215 Gbp of raw metagenomic sequencing reads from marine, soil, polar and host-associated environments, to identify DNA primes that are absent from every sequence examined in this snapshot. We then present barcodesDB, a database and software tool that enables researchers to access and query these sequences as synthetic DNA barcodes. Unlike prior approaches such as SeqCount (Hampikian and Andersen 2007), which store a full integer frequency counter per *k*-mer and are therefore limited to *k*< =13 even on a single genome, barcodesDB uses a dense bit array representation that requires only one bit per k-mer. This reduces memory requirements by at least 32-fold relative to 32-bit counter arrays, enabling prime discovery at *k*-mer lengths up to 18 across hundreds of thousands of genome assemblies. Beyond the algorithmic improvement, barcodesDB provides the most comprehensive database of DNA primes to date, a versioned record of every input the database was derived from, a publicly accessible web application, an installable implementation of the extraction pipeline (pip install barcodesDB) with which users can rebuild the index over any sequence collection of their choosing, and optional sequence-quality filters for restriction enzyme sites, homopolymers, and low-complexity sequences.

## 2. Methods

### 2.1. Genomic datasets

Reference genomes were obtained from the GenBank (Sayers *et al.* 2025) and RefSeq (Goldfarb *et al.* 2025) databases. Complete genome assemblies were downloaded across the following taxonomic groups from RefSeq and GenBank: archaea, bacteria, fungi, plants, protozoa, vertebrate mammals, other vertebrates, invertebrates, viruses, human, metagenomes, and environmental sequences. Assembly summary records for these groups were retrieved on 3 August 2026 and filtered to entries with version_status equal to “latest,” assembly_level equal to “Complete Genome,” and an available FTP download path. “Complete” is therefore the NCBI assembly_level designation, meaning that every chromosome and plasmid of the organism is represented as a single gapless sequence. This yielded 4 03 199 assembly records, of which 3 24 126 are unique sequence sets; the remaining 79 073 are GenBank (GCA) records that are byte-identical to their RefSeq (GCF) counterparts, which does not affect the result because *k*-mer presence is idempotent under duplication. The set comprises 3 24 008 GenBank and 79 191 RefSeq records and breaks down into 2 50 077 viral, 1 49 868 bacterial, 1653 archaeal, 778 fungal, 297 plant, 291 metagenome-assembled, 65 invertebrate, 61 protozoan, 55 non-mammalian vertebrate, and 37 mammalian assemblies, together with 17 records without an assigned NCBI group. It spans 53 784 distinct species and 109 620 distinct taxa and totals 1022.5 Gbp of sequence, or 739.6 Gbp once the duplicate records are excluded. The complete list of accessions, with organism name, assembly name, taxonomic group, genome size, GC content and species taxid for every record, is distributed with the database as a downloadable table. *K*-mer lengths of up to and including 18 base pairs were analyzed to identify *k*-mers absent across all inputs. The database is updated on a quarterly basis by re-running the pipeline against the latest available NCBI complete genome assemblies together with an expanded set of raw sequencing runs, and each rebuild is published under a new version number. The release described in this manuscript is v1.0.

To extend coverage beyond assembled reference genomes, we additionally indexed raw sequencing reads from 12 metagenomic runs deposited in the SRA and ENA repositories, comprising 1 72 61 60 204 reads and 215 Gbp of sequence. These runs were selected to span environments that are poorly represented among complete assemblies: human gut from six cohorts in China, Honduras and the United States (SRR16555193, SRR25477458, SRR29868879, SRR5056826, SRR11412694, SRR30767187, and SRR11412679), mouse gut (SRR28087384), soil from a global standardized survey (SRR30731270), Southern Ocean sea ice and seawater (SRR31550839), and the Tara Oceans protist size fraction sampled at 5–7 m and 30 m depth (ERR538184 and ERR1726848). FASTQ inputs are parsed as FASTQ, so quality lines are omitted and no *k*-mer is permitted to span two reads. Assemblies and reads are processed in the same pass, over 4 04 075 input files in total, and a *k*-mer observed in any input marks both itself and its reverse complement as present, so that a reported barcode is absent on both strands.

### 2.2. Data provenance and versioning

Because barcode sequences are prioritized based on their absence, the resulting prioritization is meaningful only in the context of the set of sequences from which it was derived. Each barcodesDB release therefore records its own inputs. A machine-readable changelog, served at http://barcodesdb.com/changelog.html and versioned in the code repository, captures for every release the snapshot date, the assembly-level filter applied, the number of assemblies and of unique assemblies in each taxonomic group together with their species counts, sequence volume and mean GC content, the identity, environment, platform and read volume of every sequencing run, and the resulting barcode counts at each value of *k*. A per-assembly table listing all 4 03 199 accessions is available for download from the same page. This allows a user to establish exactly which sequences a given barcode set was tested against, to identify taxonomic or environmental gaps in the current release, and to reproduce the extraction from the same inputs. The release described here, v1.0, was built from a snapshot dated 3 August 2026 and published on 4 August 2026.

### 2.3. Derivation of prime sequences

We computed barcode *k*-mers by representing the entire 4^*k* DNA *k*-mer space as a dense bit array, where each possible *k*-mer maps to a unique integer index in [0, 4^*k*). The indexing is a bijection between length-*k* strings over {A, C, G, T} and the integer range [0, 4^*k*), so each *k*-mer corresponds to exactly one bit position. *k*-mers are ordered first by GC count and then lexicographically (A < C < G < T) within each equal-GC block: the block containing exactly *g* occurrences of C or G begins at index Σ_{*h* < *g*} C(*k*, *h*)·2^*k* and holds C(*k*, *g*)·2^*k* entries. Ordering by GC count places every *k*-mer of a given GC content in a contiguous index range, so a GC-constrained query scans a slice of the array rather than all of it. A plain base-4 encoding (A = 0, C = 1, G = 2, T = 3, leftmost base most significant) is also implemented and selectable; the two are interchangeable provided the same choice is used for extraction and for querying. The bit array is initialized to all zeros, meaning “unseen”; when a *k*-mer is observed anywhere in the input database, its corresponding bit is set to 1. After processing, barcodes are defined as the *k*-mers whose bits remain 0 (i.e. sequences not observed anywhere in the dataset at the selected *k*).

*K*-mers are extracted from each input genome/contig sequence using a streaming sliding-window procedure, implemented in C++. As sequence characters are read, a rolling *k*-mer representation is maintained and updated at each new base; once at least *k* valid bases have been accumulated, the current window yields one *k*-mer. Any non-canonical character (non-ACGT) breaks the window and resets the rolling state, preventing invalid *k*-mers from being emitted. This streaming approach performs constant work per input base, avoids storing entire sequences in memory, and ensures that *k*-mer extraction is a single-pass operation over the input data.

The implementation is parallelized across CPU threads by distributing input files across a shared work scheduler: each thread processes different files independently while updating a single shared global bit array. Thread safety is ensured via atomic updates at byte granularity: setting a bit is implemented as an atomic read-modify-write OR on the containing byte, so concurrent attempts to set the same bit cannot cause data races or lost updates (the operation is idempotent). Each compute node produces its own bit array output (memory-mapped file on disk). For the full NCBI-scale run, we used 2 nodes (144 CPUs per node) to generate three independent bit arrays, and then merged them via a bitwise OR to obtain the final presence/absence array; barcodes are then obtained by scanning the merged array for zero bits (Fig. 1a). Peak memory usage scaled with *k* in close agreement with the theoretical bit array size of 4^*k* bits (Fig. 2). In contrast, the SeqCount algorithm introduced by Hampikian and Andersen (2007) uses a counter array storing one integer per *k*-mer rather than one bit, requiring orders of magnitude more memory and limiting practical operation to *k* ≤ 13 on a single genome. Our bit array representation reduces memory requirements by a factor of at least 32 relative to 32-bit counter arrays, enabling genome-scale prime discovery at *k* = 18 across hundreds of thousands of assemblies on standard HPC hardware.

**Figure 1:**
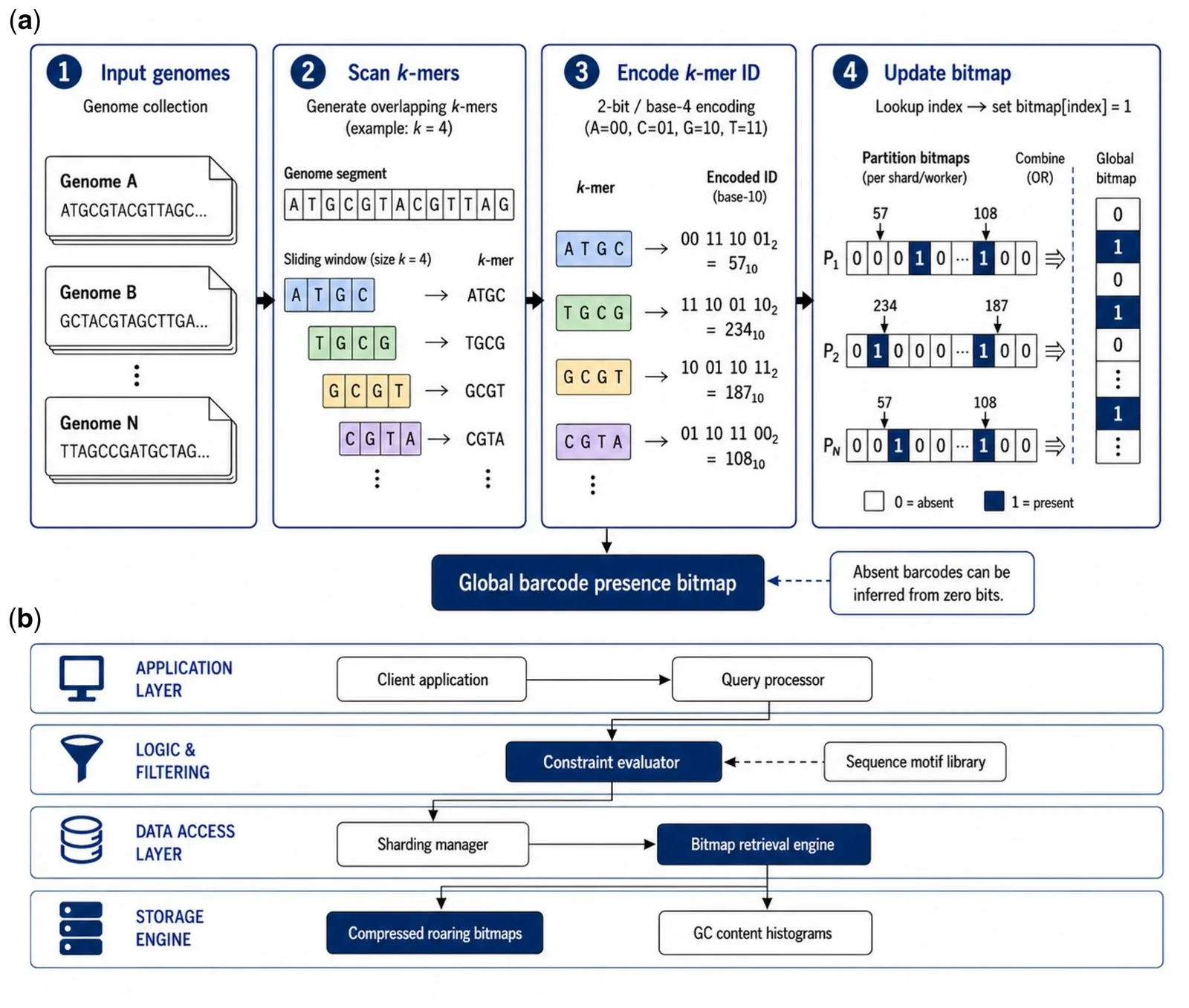
Overview of the barcodesDB extraction workflow and database architecture. (a) Schematic representation of the bitmap-based algorithm used to extract barcode sequences from a collection of FASTA files. (b) Architecture of the barcodesDB system, illustrating the organization of extracted barcode data and the database components supporting storage and query operations.

**Figure 2:**
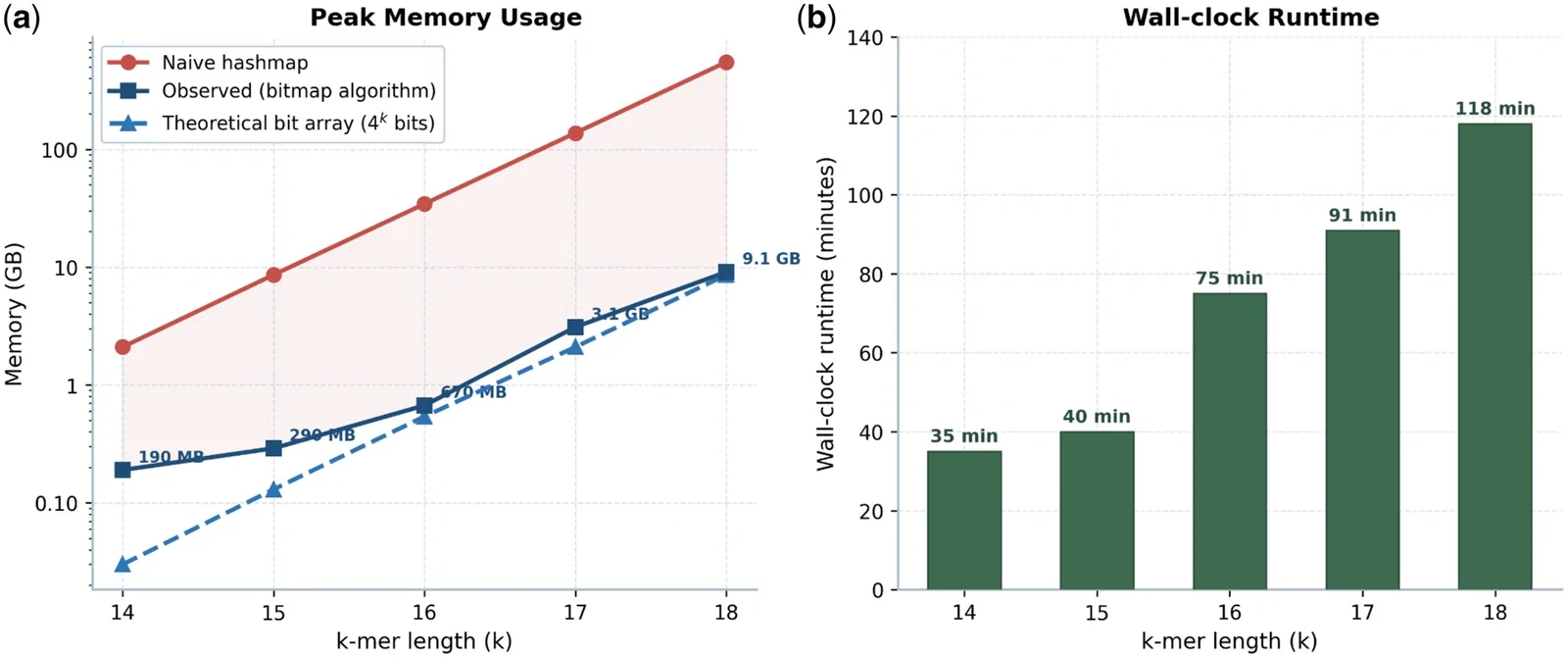
Computational performance of the bit-array pipeline across *k*-mer lengths *k* = 14–18, evaluated on 4 03 199 complete genome assemblies and 12 metagenomic sequencing runs using 2 HPC nodes (144 cores per node, aarch64 architecture). (a) Peak memory usage plotted on a log scale alongside the theoretical minimum for a dense bit array of 4^*k* bits and the estimated memory requirement of a naive hashmap storing one 8-byte entry per possible k-mer. (b) Wall-clock runtime.

Memory usage is dominated by the dense bit array, which scales deterministically with *k*. Because there are 4^*k*^ = 2^2^^*k*^ possible DNA *k*-mers and we store one bit per *k*-mer, the required memory is 4^*k*^ bits = 2^2^^*k*^^−3 ^bytes (plus negligible metadata and buffering). This dense representation is substantially more efficient than a hash map for large-scale *k*-mer presence tracking: it avoids per-entry overhead, yields predictable memory usage, improves cache locality, and supports fast sequential merging by OR. Overall, the procedure is linear in the amount of input sequence processed (streaming sliding window) plus a linear pass over the fixed-size array when enumerating unseen *k*-mers, enabling high-throughput barcode discovery on large reference collections.

### 2.4. barcodesDB database layer

The data querying layer provides two complementary capabilities over a compact presence database of natural *k*-mers.

For each input barcode (a DNA *k*-mer), we map the sequence to a unique integer index using the same GC-count-then-lexicographic encoding applied during extraction. We then check the corresponding position in a compressed bitset of observed *k*-mers to return an exact present/absent call. In parallel, nucleotide composition statistics including A, C, G, and T counts and GC percentage are computed directly from the query string and returned alongside the membership call, enabling downstream filtering and reporting without storing per-sequence annotations in the database. Batch performance is achieved through streaming input parsing and buffered output to reduce per-record overhead while preserving deterministic semantics.

For motif-based discovery searches, we enumerate barcodes from the database and return those that match in paginated results. The database is partitioned into multiple shards to allow selective loading and incremental scanning. Within each shard, we iterate over the compressed representation to list only barcodes that are present, rather than expanding the full *k*-mer space. Each candidate barcode is filtered against user-specified constraints including GC bounds and substring filtering. When enabled, the substring constraint can also accept the reverse complement of the query substring, allowing strand agnostic matching. The retrieval layer also supports returning barcodes longer than the native database length by expanding enumerated seed sequences into longer candidates and applying the same GC and substring filters at the target output length. Paginated navigation is implemented via continuation tokens that encode sufficient iteration state to resume scanning without repeating or skipping results, to achieve consistent navigation and direct jumps among already-fetched pages. Because any *k*-mer that contains a prime as a substring is itself guaranteed to be absent from the examined genomes, every extended barcode returned at lengths >18 is a valid prime; however, this set is a strict subset of all primes at that longer length, since primes of length *k* > 18 that do not contain any *k* = 16, 17, or 18 prime as a substring are not enumerated. For this reason, the *K*-mer lookup page, which performs exact present/absent calls against the underlying bitmaps, supports only *k* = 16, 17, and 18—the lengths at which we have a complete, exhaustive enumeration of primes.

### 2.5. barcodesDB web application

These query capabilities are provided through a web application consisting of a lightweight HTTP service and a browser-based interface (Fig. 1b). The server validates query parameters such as substring, reverse-complement matching, GC bounds, output length, and page size, delegates computation to the local query layer, and returns structured results annotated with sequence composition metrics (A, C, G, T counts, and GC percentage). The interface includes two primary pages. The Search barcodes page supports interactive barcode discovery by allowing users to search for barcodes that satisfy a substring constraint (optionally including the reverse complement), apply GC constraints, page through results returned from the database, and export the displayed page as a tab-delimited text file (Fig. 3a). The *K*-mer lookup page supports direct existence queries by allowing users to submit a list of candidate barcodes and returning exact present or absent calls for each query sequence, together with composition statistics and download options to export present, absent, or all queried sequences in tab-delimited format (Fig. 3b). The Generate barcodes page provides a random sequence generator that creates synthetic barcodes matching user-specified length, GC content range, substring constraint, and optional motif filters, operating independently of the database for applications requiring custom barcode design.

**Figure 3:**
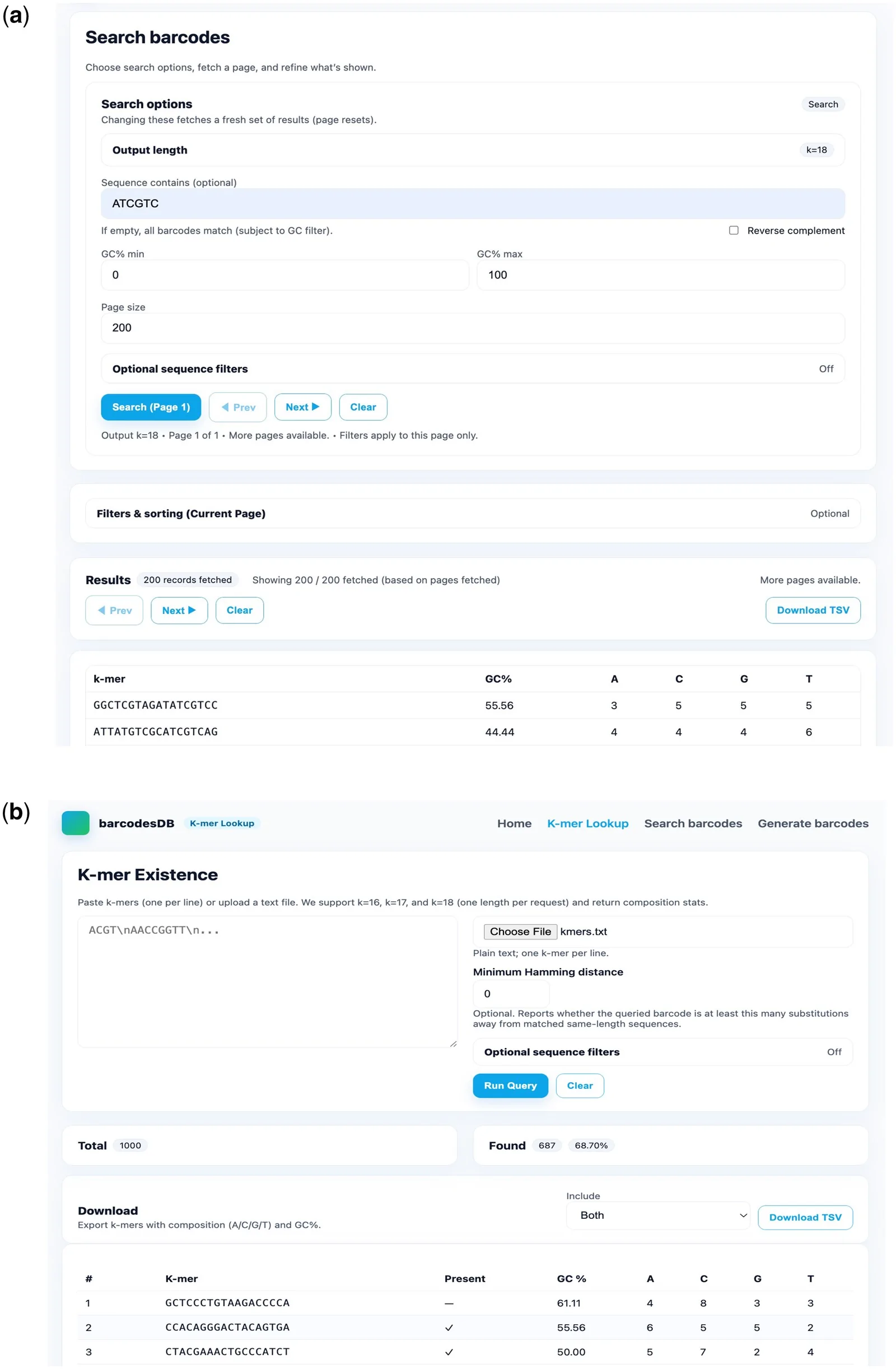
barcodesDB web application for barcode discovery and existence queries. (a) Search barcodes interface showing substring-based barcode retrieval with optional reverse-complement matching, GC constraint controls, paginated navigation, and export of displayed results with composition annotations. (b) *K*-mer lookup interface for batch existence queries, reporting exact present or absent calls per input sequence along with nucleotide composition metrics and tab-delimited export options.

### 2.6. Sequence quality filters

barcodesDB provides five categories of optional sequence-based filters that can be applied to candidate barcodes retrieved from the database. First, restriction enzyme recognition site filtering screens each barcode against a curated panel of 50 recognition sequences on both strands, excluding any barcode containing a recognition site for one or more specified enzymes; users may restrict screening to individually selected enzymes through the web interface and API, making this filter relevant for applications in which barcodes are delivered by cloning-based methods. Second, homopolymer filtering excludes barcodes containing runs of consecutive identical nucleotides exceeding a user-configurable threshold (default: runs longer than four bases, i.e. five or more identical bases in a row), which are known to reduce sequencing accuracy on short-read platforms. Third, low-complexity filtering applies Shannon entropy scoring (H = −Σ p·log₂p over A/C/G/T) to identify and exclude barcodes with insufficient nucleotide diversity, with a user-configurable minimum entropy threshold (default 1.5 bits). Fourth, tandem repeat filtering detects periodic repeat tracts and operates independently at the dinucleotide (≥6 bp; ≥3 consecutive repeats), trinucleotide (≥9 bp), and tetranucleotide (≥12 bp) levels, targeting microsatellite-like sequences that impair sequencing and amplification accuracy. Fifth, functional-motif filtering screens barcodes against a curated panel of short regulatory and signaling sequence patterns, including core promoter elements (TATA box, GC box), polyadenylation signals, splice-site-like sequences, transcription factor binding cores, and CpG dinucleotides, on both strands, which may be undesirable when barcodes are expressed or transcribed. Finally, users can also include custom motifs to filter barcodes. All filters are optional and independently configurable through the web interface and API, allowing users to apply the subset of constraints appropriate for their experimental context.

### 2.7. Minimum pairwise Hamming distance filter

To support construction of sequencing-error-robust barcode libraries, barcodesDB provides a minimum pairwise Hamming distance filter. Given a batch of candidate barcodes, the server computes all pairwise Hamming distances and annotates each barcode with its nearest-neighbor distance, the minimum Hamming distance to any other barcode in the batch. Users specify a minimum distance threshold (e.g. 2 or 3 substitutions for typical short-read error rates), and each barcode receives a pass or fail annotation accordingly. This filter integrates with the GC content and sequence quality filter pipelines, enabling users to simultaneously enforce genomic absence, sequence quality, and error-robustness constraints in a single query.

### 2.8. Implementation validation and quality control

The correctness of the barcode extraction pipeline is validated through a four-layer quality control strategy. First, exhaustive ground-truth validation is performed at *k* = 4 by independently re-implementing the base-4 encoding and comparing every one of the 4^4 = 256 bits in the production C++ bit array against a brute-force enumeration, yielding zero false-absent and zero false-present calls across all synthetic inputs. Second, distributed computation is verified by confirming that merging partial bit arrays produced by independent compute nodes via bitwise OR yields bit-for-bit identical results to a single unified scan over the same input, guaranteeing that the parallel sharding step introduces no errors. Third, the presence-to-absence inversion is confirmed to be a perfect bitwise complement, ensuring every bit position is correctly accounted for. Fourth, an orthogonal spot-check independently re-scans a random sample of source FASTA files using a pure *k*-mer extractor sharing no code path with the production pipeline, confirming that barcodes emitted by the database are genuinely absent from the input sequences. Because the encoding is purely combinatorial and parameterized only by *k*, with no heuristics, approximations, or data-dependent branches, correctness proven at *k* = 4 by exhaustive enumeration directly implies correctness at *k* = 18.

## 3. Results

We scanned 4 03 199 complete genome assemblies from RefSeq (Goldfarb *et al.* 2025) and GenBank (Sayers *et al.* 2025), spanning archaea, bacteria, fungi, plants, protozoa, vertebrate mammals, other vertebrates, invertebrates, viruses, human, metagenomes, and environmental sequences, together with 215 Gbp of raw metagenomic sequencing reads from 12 SRA and ENA runs covering marine, polar, soil and host-associated environments. Using our bit array pipeline, we identified the complete set of DNA primes (*k*-mers absent from all examined assemblies and sequencing reads, on either strand) at *k*-mer lengths 14 through 18. At *k* = 14, no prime sequences were found: the *k*-mer space (4^14 = 26 84 35 456 possible sequences) is entirely saturated by the input data. At *k* = 15 the space (4^15 = 1 07 37 41 824 possible sequences) is likewise fully saturated and no primes remain. At *k* = 16 the first primes appear, but only 4634 of the 4 29 49 67 296 possible 16-mers are absent (0.00011%), a number too small for practical barcode library construction. At *k* = 17, 43 833 856 primes were identified (0.26% of the *k*-mer space), establishing *k* = 17 as the shortest length at which a useful barcode database can be constructed, and at *k* = 18, 5 845 777 189 primes were identified (8.51%), demonstrating the rapid expansion of available barcode space with increasing *k*-mer length. All three lengths at which primes exist, *k* = 16, 17, and 18, are served through the web application, with *k* = 16 retained for completeness rather than for library design.

Wall-clock runtime and memory consumption of the complete pipeline across all 4 04 075 input files on 2 HPC nodes (144 cores per node, aarch64 architecture) are summarized in Fig. 2. Runtime grew approximately linearly with *k*, from 35 min at *k* = 14–118 minutes at *k* = 18 (Fig. 2b), reflecting the dominant cost of the streaming sliding-window *k*-mer extraction. Peak memory usage scaled deterministically with k and remained within a small constant overhead of the theoretical minimum for a dense bit array of 4^*k* bits, rising from 190 MB at *k* = 14–9.1 GB at *k* = 18 (Fig. 2a). Critically, the bit-array representation is dramatically more memory-efficient than a naive hashmap storing one 8-byte entry per possible *k*-mer: at *k* = 18 the hashmap would require approximately 550 GB of RAM, exceeding the capacity of any standard compute node, whereas our bitmap fits comfortably in under 10 GB. This memory advantage widens with *k*, from ∼11-fold at *k* = 14 to ∼60-fold at *k* = 18 (Fig. 2a), and is what enables exhaustive enumeration of primes at *k* = 18 across hundreds of thousands of genome assemblies and hundreds of gigabases of raw sequencing reads. It also goes substantially further than the counter-array implementation of Hampikian and Andersen (2007), which stores a full integer per *k*-mer (a 32-fold overhead versus our single-bit representation) and is limited in practice to *k* ≤ 13 on a single genome.

The barcodesDB web application provides two complementary interfaces for accessing these results. The Search Barcodes page enables users to retrieve barcodes matching user-defined constraints including GC content bounds and substring requirements, with optional filters for restriction enzyme recognition sites, homopolymer runs, and low-complexity sequences. The *K*-mer Lookup page allows users to query whether candidate sequences are present or absent across all examined genomes, returning exact membership calls along with nucleotide composition statistics (Fig. 3).

## 4. Discussion

Here, we present barcodesDB, a new database of synthetic DNA barcodes designed to be absent from natural sequences. By leveraging the concept of DNA primes, sequences absent from all genomes examined (Hampikian and Andersen 2007), we were able to identify the set of the shortest DNA sequences completely missing across 4 03 199 complete organismal genome assemblies spanning the tree of life, together with 215 Gbp of raw metagenomic sequencing reads, as of the most recent database update. barcodesDB provides barcodes with maximal specificity, minimizing cross-reactivity, internal redundancy, and unintended biological interactions.

The use of barcodesDB is to generate synthetic barcode libraries for settings in which barcodes are introduced as engineered sequences and are not expected to occur naturally. These include high-throughput sequencing, single-cell and spatial genomics, lineage tracing, CRISPR-based screens, massively parallel reporter assays, synthetic biology, molecular provenance tracking, forensic tagging, anti-counterfeiting technologies, CRISPR landing-pad design, and DNA-based information storage systems capable of encoding metadata into DNA molecules or physical materials (Meyer and Kircher 2010, Kircher *et al.* 2012, Goldman *et al.* 2013). The optional sequence quality filters are deliberately application-dependent: restriction enzyme site exclusion is critical when barcodes are cloned via restriction-ligation, but irrelevant in purely synthetic delivery contexts; homopolymer and low-complexity filters are most important for short-read sequencing applications but may be unnecessary for long-read or hybridization-based detection (Johnson *et al.* 2023). Users are encouraged to enable only the filters appropriate for their experimental workflow. Once delivered, barcodes are integrated into experimental constructs at defined positions flanked by known primer or adapter sequences, and their detection within sequencing reads is handled by standard downstream demultiplexing tools such as cutadapt or starcode, which extract barcodes based on flanking-sequence context. The absence-from-genomes guarantee provided by barcodesDB additionally ensures that any read segment matching a barcode can be unambiguously attributed to the synthetic construct rather than to endogenous sequence, eliminating a major source of false-positive identification.

Several limitations of this work should be acknowledged. Absence from the collection indexed here is not the same as absence from nature, and the guarantee barcodesDB provides is the operational one: a barcode is absent, on either strand, from a stated, versioned and reproducible body of sequence data whose full contents are published with each release. Although the current release encompasses 4 03 199 complete genome assemblies and 215 Gbp of raw sequencing reads, it does not capture the full breadth of biological sequence diversity. For the majority of species we do not have complete genome assemblies to date (Lawniczak *et al.* 2025), meaning that barcodes classified as absent may exist in genomes not yet represented in public repositories. Also, genetic variation within species, including polymorphisms, somatic mutations, and structural variants among individuals, may give rise to sequences not captured by reference genomes (Chan *et al.* 2025). In addition, the inputs are restricted to assemblies at NCBI assembly level “Complete Genome” together with the raw sequencing runs listed in the Methods, whereas the Nucleotide collection also contains draft assemblies, scaffolds, contigs, and WGS records of any deposition date; a barcode absent from our inputs may therefore occur in a sequence record of one of those classes. Collectively, these factors mean that while barcodesDB substantially reduces the probability of off-target interactions, it cannot entirely eliminate this possibility. To allow users to verify their selected barcodes against the most current sequence data between database updates, the barcodesDB web application provides a BLAST batch (nr/nt) button that submits all returned barcode sequences directly to NCBI BLAST against the Nucleotide collection (nr/nt) database, which is broader than the assembly-level set from which the barcodes are derived. This enables independent verification that a candidate barcode remains absent from newly deposited genomes that have not yet been incorporated into barcodesDB. Because nr/nt includes draft and WGS records that lie outside our assembly-level scope, this route also allows a candidate to be screened against sequence classes the database does not index. Furthermore, while all barcodes in barcodesDB are guaranteed to be exact primes (i.e. absent from all examined assemblies), we do not currently compute distances between barcodes and natural sequences; near-match interactions with genomic sequences differing by one or a few nucleotides are therefore not screened out, and this remains an important direction for future work, particularly for applications where partial hybridization is a concern. Finally, absence from sequenced data is a statement about occurrence. A barcode that occurs nowhere in the indexed collection cannot be mistaken for an endogenous sequence during read mapping or alignment, and has no perfect complement in that collection, but it may still be bound by proteins, act as a cryptic regulatory or splice element, or otherwise interact with the cellular environment of an organism into which it is introduced. Primality eliminates sequence identity as a source of ambiguity; it does not, however, guarantee biological neutrality. Users can additionally filter sequences based on custom sets of sequence motifs.

Future work will focus on several key areas. Experimental validation of barcode performance across diverse biological contexts will be useful to confirm the specificity of this approach. Additionally, as genomic databases continue to expand, we will update barcodesDB on a quarterly basis to incorporate newly sequenced genomes, which we anticipate will further refine the barcode set and reduce the likelihood of off-target effects. Furthermore, at *k* = 14 and *k* = 15 no prime sequences exist across the current database, and at *k* = 16 only 4634 remain, while at *k* = 17 the number rises to 4 38 33 856 confirming that *k* = 17 represents the practical minimum length for a useful barcode database defined against the full tree of life. That minimum applies to primes defined against the whole of the indexed collection, and is not a limit on the approach itself. Barcode space appears abruptly as *k* grows, and where the transition falls depends entirely on how much sequence is indexed, so a database restricted to a single organism or a single environment leaves usable barcodes at substantially shorter lengths. To make this accessible, the extraction pipeline is now distributed as an installable Python package (pip install barcodesDB). It accepts FASTA and FASTQ input, plain or gzipped, builds a presence bitmap at any value of k over any collection of genomes, metagenomes or raw reads the user supplies, and provides commands to report the available barcode space, to list barcodes subject to GC content, homopolymer, entropy, restriction site and motif constraints, and to query individual candidates against a database. Users who require primes defined against a specific organism, against their own laboratory assemblies, or against the environment in which they work, or who require barcodes shorter than *k* = 16, can therefore run the same algorithm locally on a small set of genomes, which is the common situation in assay barcode design.

The web application does not currently allow a user to assemble a custom set of genomes and metagenomes from public repositories and run the extraction on demand, since this requires server-side download and indexing of user-specified data; we plan to add this capability, along with user-selectable input sets, in a future version. We are committed to the long-term maintenance and continued development of barcodesDB as a resource for the research community.

## Data Availability

All relevant code for the extraction of the barcode prime sequences can be found at: https://github.com/Georgakopoulos-Soares-lab/barcodes_extraction. The extraction tool is also available as an installable Python package from PyPI https://pypi.org/project/barcodesdb/1.0.0/. The per-assembly provenance table and the versioned release changelog recording every input used to build each release are available at http://barcodesdb.com/changelog.html. All relevant code for the database can be found on GitHub at this link: https://github.com/Georgakopoulos-Soares-lab/barcodesDB
